# NADPH oxidase 4 deficiency increases tubular cell death during acute ischemic reperfusion injury

**DOI:** 10.1038/srep38598

**Published:** 2016-12-07

**Authors:** Stellor Nlandu-Khodo, Romain Dissard, Udo Hasler, Matthias Schäfer, Haymo Pircher, Pidder Jansen-Durr, Karl Heinz Krause, Pierre-Yves Martin, Sophie de Seigneux

**Affiliations:** 1 Laboratory of Nephrology, Department of Medicine Specialties and PHYM department, University Hospital and University of Geneva, Switzerland; 2 Department of Biology, Institute of Molecular Health Sciences, ETH Zurich, Switzerland; 3 Universität Innsbruck, Institute for Biomedical Aging Research, Innsbruck, Austria; 4 Department of Pathology and Immunology, University of Geneva, Switzerland.

## Abstract

NADPH oxidase 4 (NOX4) is highly expressed in kidney proximal tubular cells. NOX4 constitutively produces hydrogen peroxide, which may regulate important pro-survival pathways. Renal ischemia reperfusion injury (IRI) is a classical model mimicking human ischemic acute tubular necrosis. We hypothesized that NOX4 plays a protective role in kidney IRI. In wild type (WT) animals subjected to IRI, NOX4 protein expression increased after 24 hours. NOX4 KO (knock-out) and WT littermates mice were subjected to IRI. NOX4 KO mice displayed decreased renal function and more severe tubular apoptosis, decreased Bcl-2 expression and higher histologic damage scores compared to WT. Activation of NRF2 was decreased in NOX4 KO mice in response to IRI. This was related to decreased KEAP1 oxidation leading to decreased NRF2 stabilization. This resulted in decreased glutathione levels. *In vitro* silencing of NOX4 in cells showed an enhanced propensity to apoptosis, with reduced expression of NRF2, glutathione content and Bcl-2 expression, similar to cells derived from NOX4 KO mice. Overexpression of a constitutively active form of NRF2 (caNRF2) in NOX4 depleted cells rescued most of this phenotype in cultured cells, implying that NRF2 regulation by ROS issued from NOX4 may play an important role in its anti-apoptotic property.

NADPH oxidases are enzymes producing free radicals as their main product^1^. Their respective role is determined by their regulation and localization. NADPH oxidase type 4 (NOX4) is highly expressed in the kidney, mostly in the highly metabolic proximal tubular compartment^2^. In contrast to other NOXs producing superoxide, NOX4’s final product is mainly hydrogen peroxide, probably due to its structure allowing dismutation of superoxide^3^. NOX4 activity appears to be constitutive and its role in diseases is very controversial^4^. Previous research demonstrated that NOX4 expression was increased in various types of kidney disease, such as diabetic and hypertensive nephropathies, where it may play a deleterious role^5^,^6^,^7^. In contrast, more recent work using NOX4 knockout (KO) animals showed that this enzyme may have a crucial role in the regulation of anti-oxidant and micro-vascularization pathways in the cardiovascular system^8^,^9^,^10^. In the lung, NOX4 appears to have an anti-apoptotic effect in lung fibroblasts, resulting in a profibrotic phenotype^11^. In the kidney, we and others have demonstrated that the absence of NOX4 under condition of tubular lesion induced by urinary obstruction enhanced fibrosis formation and tubular mass loss^12^,^13^. In contrast, glomerular lesions of diabetic nephropathy appear either unchanged or rescued by NOX4 deletion, depending on the model used^12^,^14^,^15^. These seemingly contradictory results indicate that NOX4 possesses a cell-specific role, with a physiological role in compartments where it is highly expressed such as in tubular cells. The role of NOX4 therefore remains to be determined both in kidney physiology and pathology in order to better target this protein at the therapeutic level.

The Nuclear factor (erythroid-derived 2)-like 2 (NRF2) system is a multifaceted cell defense pathway^16^. NRF2 is a transcription factor sequestered in the cytoplasm by its inhibitory protein KEAP1, which mediates its constant proteasomal degradation. Upon oxidation of specific cysteine residues of KEAP1, NRF2 is stabilized and translocates to the nucleus where it heterodimerizes with cofactors and induces the transcription of various cytoprotective genes by binding to antioxidant responsive elements (AREs)^17^. In the kidney, NRF2 appears to play an important role both in acute kidney injury (AKI) and in fibrosis formation^18^,^19^,^20^. Indeed, NRF2 target genes are induced in IRI and NRF2 knockout animals display higher tubular injury scores that can be reversed by gluthatione or N-acetylcysteine. In human chronic kidney disease, NRF2 activation was to be promising for diabetic nephropathy treatment, but revealed associated with elevated cardiovascular events^21^.

AKI is defined by a rapid loss of kidney function, resulting in increased levels of nitrogenous waste products and dysregulation of volume and electrolyte homeostasis^22^. Although usually in part reversible, this syndrome is associated with an elevated morbidity and mortality in patients, and most frequently associated to ischemia reperfusion injury. In addition, AKI may also promote progression of chronic kidney disease^23^. No therapy currently exists to prevent or cure AKI and the role of oxidative stress in its pathogenesis is debated. Indeed, enhanced oxidative stress was described after IRI in animal models^24^. However, baseline ROS production may also be protective in IRI and increased baseline superoxide production has been described to improve resistance to IRI in mice^25^,^26^. The role of NOX4 in kidney IRI is not known. In the heart, NOX4 deletion does not protect from IRI and may even have a deleterious effect on microvascularisation when combined with NOX2 deletion^27^,^28^.

In this manuscript, we demonstrate that NOX4 KO mice are more prone to kidney tubular injury during ischemia reperfusion. This is due to enhanced propensity to tubular cell apoptosis probably via regulation of NRF2 and modulation of cellular glutathione content, Bcl-2 expression and mitochondrial function.

## Results

### NOX4 expression increases 24 hours after IRI

**Figure 1:**
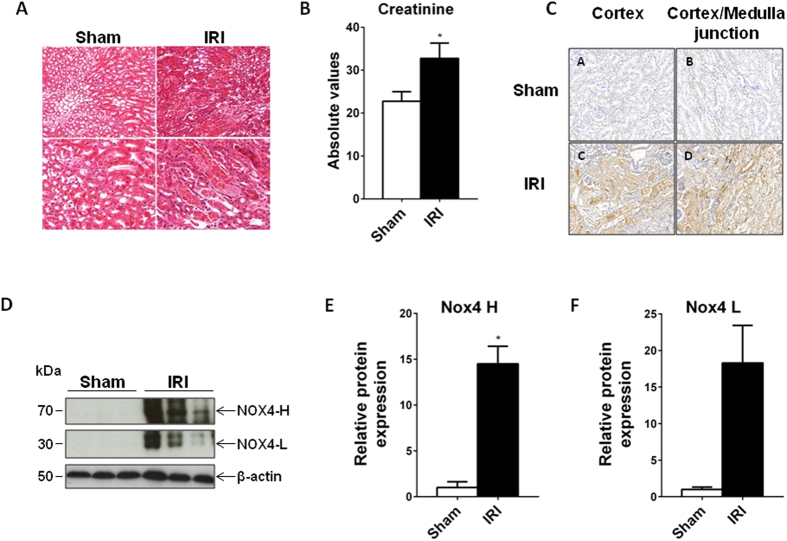
NOX4 expression is upregulated after ischemia reperfusion injury (IRI). (**A**) Representative images of cortico-medullary junction Haematoxylin Eosin staining depicting tubular injury are shown for Sham and 22 minutes ischemia reperfusion (IR) operated animals 24 hours after reperfusion. Scale bar 100 μm upper and 50 μm lower panels. (**B**) Serum creatinine measurement performed on sham and IR animals (Sham, n = 7; IR, n = 6) at 24 hours after IR. Results are expressed as absolute creatinine values in umol/l ± SEM, ns p > 0.05, *p < 0.05. (**C**) Representative images of NOX4 immunostaining performed in Sham (Sham, up) and IRI (IRI, down) kidneys from WT mice in the cortex and the cortico-medullar (CM) jonction. (**D–F**) NOX4 Western blot analysis performed on kidney cortex protein samples from sham and IR operated animals for the high (H) and low (L) molecular band. Representative picture and Western blot densitometric quantification are shown (Sham, n = 6; IR n = 5). Results are expressed as the mean of individual densitometric values over the mean densitometric value obtained in sham operated animals ± SE, ns p > 0.05 and *p < 0.05.

WT mice subjected to 22 minutes of warm ischemia displayed tubular necrosis as opposed to sham-operated littermates 24 hours after reperfusion as assessed by histology (Fig. 1A). Histological kidney injury correlated with increased serum creatinine levels (Fig. 1B). Immunochemistry showed an increase in NOX4 expression, particularly in injured proximal tubular cells (Fig. 1C), which was confirmed by Western blot analysis (Fig. 1D–F). The specificity of NOX4 antibody was demonstrated in NOX4 KO mice (Supp. Fig. 1).

### NOX4 KO mice display increased tubular apoptosis and creatinine levels under IRI

**Figure 2:**
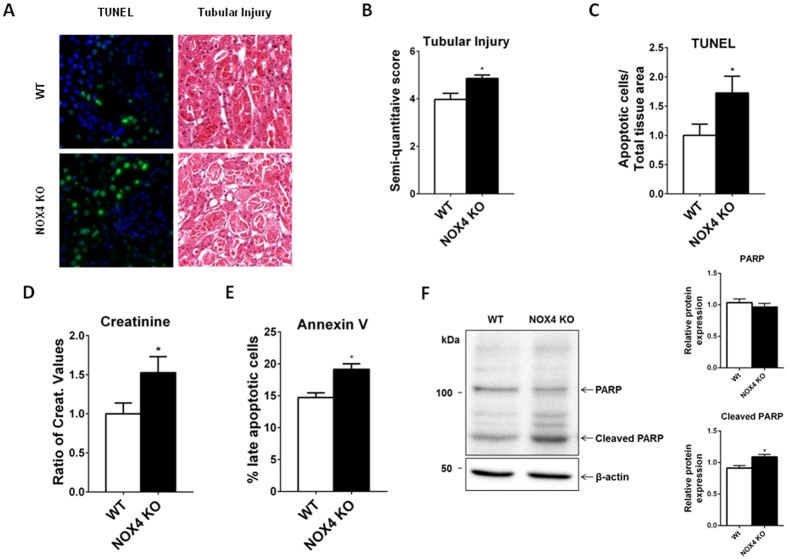
Tubular apoptosis and injury is increased in NOX4 KO mice compared to WT after IRI (A–E). (**A**) Representative images of renal cortex TUNEL/DAPI staining and cortico-medullary Haematoxylin Eosin staining depicting tubular injury in WT and NOX4 KO mice 24 hours after IR are shown. Scale bar 50 μm. (**B**) Quantification of renal cortico-medullary junction injury score in NOX4 KO mice compared to WT (WT, n = 10; NOX4 KO, n = 9) after ischemia reperfusion. Results are expressed as the mean of arbitrary values ± SEM, ns p > 0.05, *p < 0.05. (**C**) Quantification of TUNEL staining of apoptotic cells in renal cortex of NOX4 KO mice compared to those of their WT littermates (WT, n = 10; NOX4, n = 9). Results are expressed as the mean ratio of the density of TUNEL positive cells measured in each sample over the mean density of TUNEL positive cells measured in WT ± SEM, ns p > 0.05, *p < 0.05. (**D**) Serum creatinine measurement performed on WT and NOX4 KO mice (WT, n = 10; NOX4 KO, n = 9) after IRI. Results are expressed as relative values of serum creatinine of NOX4 KO versus WT in different experiments in mean ± SEM, ns p > 0.05, *p < 0.05. (**E**) AnnexinV/PI assay analysis of cell apoptosis in MEF cells derived from WT and NOX4 KO mice. Results are expressed as mean percentage of late apoptotic cells (cells positive for both Annexin V and PI ± SEM, (n = 3), ns p > 0.05, *p < 0.05. (**F**) PARP and Cleaved PARP Western blot analysis performed on freshly isolated renal cortical tubule suspensions protein samples from WT and NOX4 KO. Representative picture and Western blot densitometric quantification are shown (WT n = 3; KO n = 3). Results are expressed as the mean of individual densitometric values over the mean densitometric value obtained in WT animals ± SE, ns p > 0.05 and *p < 0.05.

We previously demonstrated that NOX4 KO animals do not display enhanced kidney tubular cell apoptosis at baseline, but were more susceptible to tubular injury during unilateral uretral obstruction (UUO)^13^. In order to test a protective role of NOX4 against apoptosis in IRI, tubular apoptosis was assessed by TUNEL assay 24 hours after 22 minutes bilateral IRI in WT and NOX4 KO mice (Fig. 2A). Tubular cell apoptosis in the cortex and outer medulla was more pronounced in NOX4 KO animals compared to their WT littermates (Fig. 2B). In addition, tubular injury score quantified at the corticomedullary junction was higher in NOX4 KO animals (Fig. 2C), in line with increased serum creatinine levels (Fig. 2D). Tubular cells depleted of NOX4 display enhanced apoptosis via the classical pathway^13^. In parallel, NOX4 KO mouse embryonic fibroblasts (MEF) cells derived from our animals displayed an increased late apoptosis *in vitro* compared to WT MEF as assessed by Annexin V/PI assay (Fig. 2E). In addition, enhanced PARP cleavage was observed in NOX4 KO MEF cells, as previously observed in kidney tubular cells depleted with NOX4^13^, both at baseline and in condition of hypoxia (Supp. Fig. 2). Finally, since no reliable proximal derived tubular cell line exists, we used kidney tubule suspension issued from both the cortex of both WT and NOX4 KO mice, therefore containing a majority of proximal tubules. During preparation, suspensions are exposed to some degree of stress and hypoxia and we clearly observed a decrease of the full length isoform of PARP and an increase of its cleaved isoform in the tubules suspension from the NOX4 KO mice compared to WT, implying enhanced apoptosis in the absence of NOX4 (Fig. 2F). The cortical suspensions used here are mainly representative of proximal tubular cells found in mice kidney as confirmed by high NaPi-IIa protein expression. The lack of NOX4 in our suspension issued from KO mice was also confirmed by western-blot (Supp. Fig. 3). Thus we observed enhanced apoptosis of tubular cells deficient in NOX4 *in vivo, in vitro* in a tubular cell line depleted with NOX4, in NOX4 deficient MEFs, as well as in tubule suspension of proximal tubular cells issued from mouse kidneys. We thereby confirm that NOX4 depletion increases apoptosis in different cell lines, including primary suspensions of tubular cells. In order to further study the molecular mechanisms of these observations, we further used both tubular cell lines depleted in NOX4 and MEF cells from NOX4 KO mice.

### NOX4 depletion decreases the expression of Bcl-2

**Figure 3:**
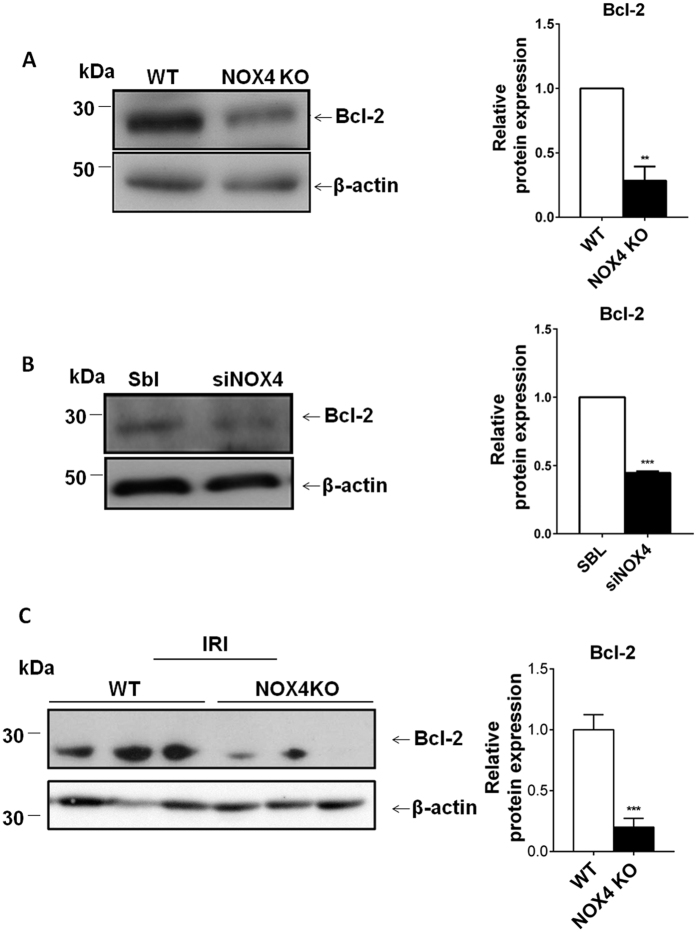
NOX4 depletion decreases the expression of the antiapoptotic factor Bcl-2. Representative Western blot of Bcl-2 protein expression in MEFs issued from WT and NOX4 KO mice (**A**) and densitometric quantification are shown. Results are expressed as the mean of densitometric values over the mean value obtained in WT cells ± SEM, (n = 3), ns p > 0.05, *p < 0.05, **p < 0.005. (**B**) Western blot analysis of Bcl-2 performed on mCCD cells transfected or not with siRNA targeting NOX4. Representative image and Bcl-2 expression densitometric quantification are shown. Results are expressed as the mean of densitometric values over the mean value obtained in mCCD_cl1_ cells transfected with scramble RNA ± SEM, (n = 3), (**C**) Representative Western blot of Bcl-2 protein expression in WT and NOX4 KO mice subjected to IRI and Bcl-2 expression densitometric quantification. Results are expressed as the mean of densitometric values over the mean value obtained in WT, (WT, n = 5, NOX4 KO, n = 6) (**F**) p > 0.05, *p < 0.05, **p < 0.005, ***p < 0.0005.

Bcl-2 is a major and well characterized anti-apoptotic factor. We hypothesize that NOX4 deletion may induce apoptosis through decreased Bcl-2 expression. *In vitro* Bcl-2 expression was decreased in NOX4 KO MEF cells (Fig. 3A) and in mCCD_cl1_ cells transfected with a siRNA targeting NOX4 (Fig. 3B), respectively in comparison to WT MEF and scrambled transfected mCCD_cl1_ cells, likely participating to the enhanced apoptosis observed in these two cell lines. Bax, another pro-apoptotic protein, was not regulated. Downstream proteins such as caspase 3 appeared down-regulated, in line with Bcl-2 regulation and enhanced apoptosis (Supp. Fig. 4). Finally, in mice subjected to IRI, Bcl-2 protein expression was largely decreased in NOX4 KO mice compared to WT, which presumably is at least partially responsible for to the increased apoptosis and tubular injury observed in these mice (Fig. 3C).

### NRF2 and NRF2 target genes are down-regulated in NOX4 KO mice subjected to IRI

**Figure 4:**
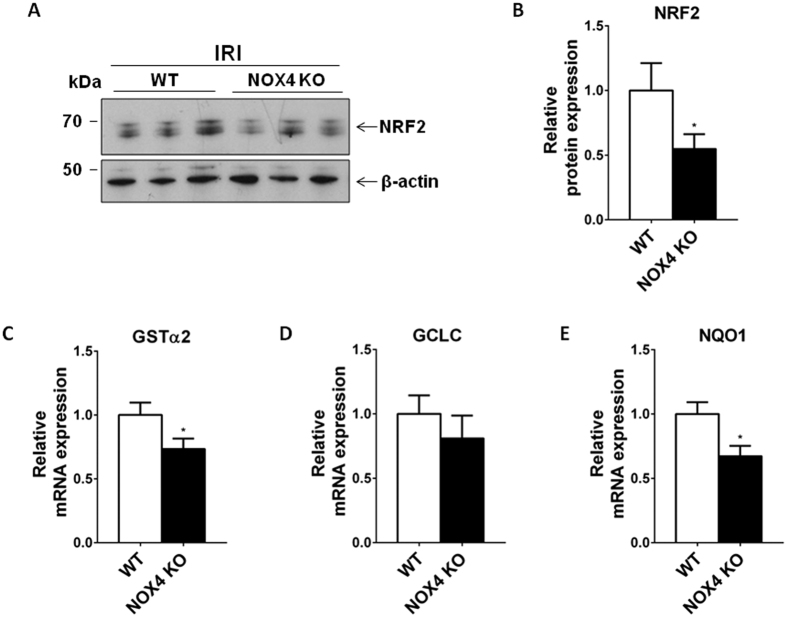
NRF2 and NRF2 target genes expressions are downregulated in NOX4 KO mice under IRI. (**A,B**) Western blot analysis of NRF2 performed on kidney cortex from NOX4 and WT mice after IR. Representative picture and densitometric quantification (WT, n = 3; NOX4 KO, n = 5) of Western blots are shown. Results are expressed as the mean of individual densitometric values of the mean of densitometric value obtained in WT animals ± SEM, ns p > 0.05, *p < 0.05. (**C–E**) Real time PCR analysis of GSTα2 (**C**), GCLC (**D**) and NQO1 (**E**) mRNA expressions performed in NOX4 KO and WT animals (WT, n = 10; NOX4, n = 9). Results are expressed as relative expression compared to WTs ± SEM, ns p > 0.05, *p < 0.05.

We previously observed that NOX4 KO mice display decreased baseline NRF2 expression^13^. Bcl-2 expression is both regulated by NRF2 as well as by KEAP1^29^,^30^,^31^. We hypothesized that NRF2 down-regulation was also observed after IRI and may participate to enhanced tubular apoptosis in NOX4 KO mice. The NRF2 transcription factor and its target genes were down-regulated in NOX4 KO animals compared to their WT littermates, as assessed by Western blot and real time PCR analysis after IRI (Fig. 4A–E). We previously demonstrated that NRF2 protein and target gene expression were down-regulated in tubular cells treated with interfering siRNA targeting NOX4^13^. In order to use a second cellular model to study the pathway of this regulation, we verified that NOX4 KO MEF cells also showed decreased NRF2 expression as well as target gene expression (GSTα2 (Gluthation-s-transferase), GCLC (Glutamate—cysteine ligase catalytic subunit)) compared to WT cells (Supp. Fig. 5).

### NOX4 regulates NRF2 by modifying KEAP1 oxidation

**Figure 5:**
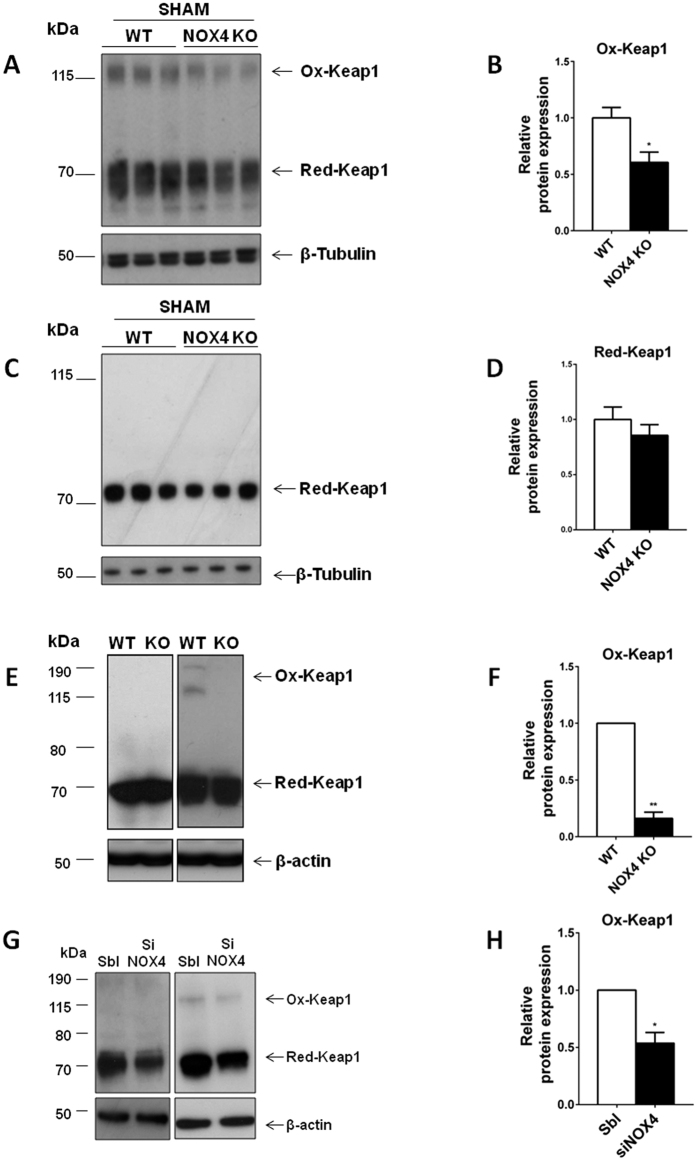
NOX4 regulates NRF2 by modifying KEAP1 oxidation. (**A–D**) Western blot analysis of KEAP1 oxidation performed on kidney cortex from WT and NOX4 KO sham mice, in non-reducing and reduced conditions. Representative images (**A,C**) and high molecular weight oxidized KEAP1 densitometric quantifications (WT = 6 and NOX4KO = 6) (**B,D**) are shown respectively in non-reduced (**A,B**) and reduced conditions (**C,D**). Results are expressed as the mean of individual densitometric values over the mean of densitometric value obtained in WT animals ± SEM, ns p > 0.05, *p < 0.05, (WT n = 6 and NOX4 KO n = 6). (**E,F**) Western blot analysis of KEAP1 oxidation performed on WT and NOX4 KO MEF cells in non-reduced and reduced conditions. Representative image (**E**) and high molecular weight oxidized KEAP1 densitometric quantifications (**F**) are shown. Results are expressed as the mean of densitometric values over the mean of densitometric value obtained in WT MEF cells ± SEM, (n = 3) ns p > 0.05, *p < 0.05, **p < 0.005. (**G,H**) Western blot analysis of KEAP1 oxidation performed on mCCD_cl1_ cells transfected or not with a siRNA targeting NOX4 under non-reducing and reducing conditions. Representative image (**G**) and high molecular weight oxidized KEAP1 densitometric quantifications (**H**) are shown. Results are expressed as the mean of densitometric values over the mean of densitometric value obtained in scramble RNA transfected mCCD_cl1_ cells ± SEM, (n = 3), ns p > 0.05, *p < 0.05.

We further tried to decipher how NOX4 regulates the NRF2 pathway *in vivo* and *in vitro*. KEAP1 is the main regulator of NRF2 in the cytosol. KEAP1 oxidation can be assessed under non-reducing conditions^32^. KEAP1 oxidation as well as expression was assessed by Western blot under non-reducing and reducing conditions in WT and NOX4 KO mice as well as in cell lines (Fig. 5A,C,E,G). KEAP1 oxidation was decreased in NOX4 KO animals compared to their WT littermates as assessed by the appearance of high molecular weight oxidized KEAP1 band under non-reducing conditions (Fig. 5A,B), whereas total KEAP1 expression was unchanged (Fig. 5C,D). KEAP1 oxidation was also decreased in NOX4 KO MEF cells compared to WT MEFs (Fig. 5E,F) as well as in mCCD_cl1_ cells transfected with siRNA targeting NOX4 in comparison to scramble RNA transfected cells (Fig. 5G,H). We further performed immunoprecipitation followed by western blotting to determine whether KEAP1 and NOX4 physically interact. There was no evidence for a physical interaction between NOX4 and KEAP1 (Supp. Fig. 6), although the Western blot data obtained for KEAP1 were difficult to interprete, since KEAP1 co-migrates in SDS-PAGE with the immunoglobulin heavy chain (Supp. Fig. 6). These data suggest that NOX4 may regulate NRF2 activity by altering KEAP1 oxidation status. Regulation of the NRF2-Keap pathway may lead to modulation of pro-inflammatory genes^33^. Coherently, using kidney tissue from both WT and KO NOX4, we observed that IRI induced expression of pro-inflammatory genes, that was even more pronounced in NOX4 KO mice compared to WT (Supp. Fig. 7).

### NOX4 regulates intracellular glutathione synthesis

**Figure 6:**
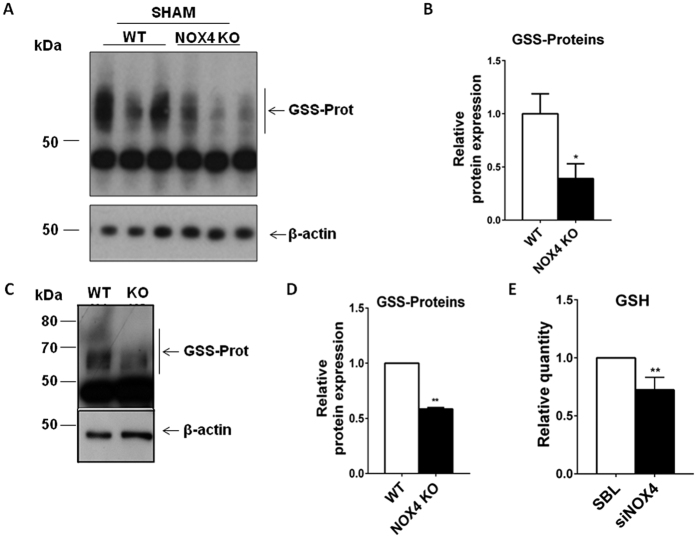
Protein glutathionylation and glutathione amount are decreased under NOX4 deficiency. (**A,B**) Western blot analysis of protein glutathionylation performed on kidney cortex from WT and NOX4 mice in non-reduced conditions. Representative image (**A**) and glutathionylated proteins densitometric quantification (**B**) are shown. Results are expressed as the mean of densitometric values over the mean of densitometric value obtained in WT ± SEM, ns p > 0.05, *p < 0.05 (WT, n = 6, NOX4 KO, n = 5). (**C,D**) Western blot analysis of protein glutathionylation performed on MEF cells from WT and NOX4 KO mice under non-reducing conditions. Representative image (**C**) and glutathionylated proteins densitometric quantification (**D**) are shown. Results are expressed as the mean of densitometric values over the mean of densitometric value obtained in WT cells ± SEM, (n = 3), ns p > 0.05, *p < 0.05, **p < 0.005. (**E**) Glutathione (GSH) amount measurement performed on mCCD_cl1_ cells transfected or not with a siRNA targeting NOX4. Results are expressed as ratio of relative quantity over the mean value obtained in WT or scramble RNA transfected mCCD_cl1_ cells ± SEM, (n = 3), ns p > 0.05, *p < 0.05.

NRF2 regulates the antioxidant glutathione pathway. Glutathione is an important tripeptidic cofactor involved in the cytoprotection against oxidative and xenobiotics stresses. Protein glutathionylation decreases in NOX4 KO animals and MEF cells in comparison respectively to their WT littermates (Fig. 6A,B) and WT derived MEF cells (Fig. 6C,D). Total glutathione amount as measured by a luciferase kit decreases in mCCD_cl1_ cells treated with an interfering RNA targeting NOX4 in comparison to scramble treated cells (Fig. 6E). This observation is consistent with mRNA expression of enzymes involved in glutathione biosynthesis previously demonstrated to be down-regulated in the absence of NOX4 in mCCD_cl1_ cells^13^ as well as in MEF cells (Supp. Fig. 5).

### Mitochondrial integrity is altered by NOX4 depletion

**Figure 7:**
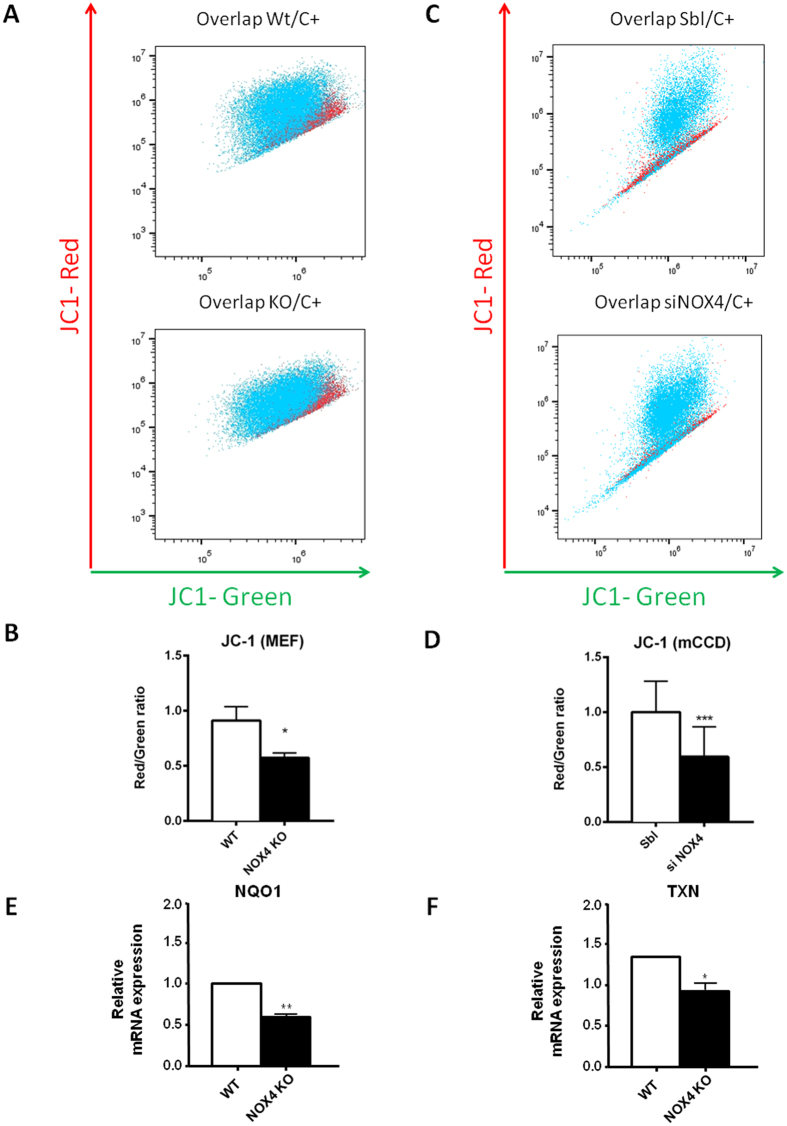
Mitochondrial stability is modified by NOX4 depletion. MEF and mCCD_cl1_ mitochondrial membrane potential was assessed by JC-1 assay based on a dye exhibiting a potential dependent accumulation in mitochondria indicated by a fluorescence emission shift from red to green. Fluorescence shift is more important in KO or silenced NOX4 cells than Wt cells. Representative quantification (**A,B**) of JC-1 in WT and MEF KOs are shown. Results are expressed as red/green ratio ± SEM, (n = 3), ns p > 0.05, *p < 0.05. Representative quantification (**C,D**) of JC-1 in Sbl and siNOX4 mCCD_cl1_ are shown. Results are expressed as red/green ratio ± SEM, (n = 3), ns p > 0.05, *p < 0.05. (**E,F**) Real time PCR analysis of NQO1 (**B**) and Thioredoxin (TXN) (**C**) performed on MEF cells derived from WT and NOX4 KO mice. Results are expressed as relative expression compared to WT cells ± S EM, (n = 3), ns p > 0.05, *p < 0.05, **p < 0.005.

Several studies reported mitochondrial or ER NOX4 localization without a clear insight into its role^34^,^35^,^36^. Mitochondrial glutathione depletion may lead to enhanced ROS mitochondrial production. Bcl-2 and NRF2 expression also affects mitochondrial membrane function^37^,^38^. We therefore assessed mitochondrial potential as a read out for mitochondrial health. MEF mitochondrial membrane potential was assessed by JC-1 assay, based on a dye exhibiting a potential dependent accumulation in mitochondria indicated by a fluorescence emission shift from red to green. NOX4 KO MEF cells displayed a decrease of mitochondrial membrane potential compared to the WT MEF cells (Fig. 7A,B), indicating instability of mitochondrial membranes in the absence of NOX4. In mCCD_cl1_ cells transfected with a siRNA targeting NOX4, NOX4 depletion induces a basal decrease of mitochondrial membrane potential compared to scramble cells (Fig. 7C,D). We further assessed different subunits of the NADH dehydrogenase (or mitochondrial complex I). We observed no downregulation of subunits tested in Complex I (NDUFS1, NDUFA9, NDUFV1), complex III (MTCO1) and IV (UQCRQ) in MEF cells (Supp. Fig. 8), nor in mCCD cells (data not shown). NADPH dehydrogenase quinone 1 (NQO1) and thioredoxin 2 (TXN), two major mitochondrial ROS scavengers, were also down-regulated in NOX4 KO MEF cells (Fig. 7E,F) as well as in mCCD_cl1_ cells^33^, probably also contributing to this mitochondrial dysfunction^39^.

### Overexpression of active NRF2 protein partly reverses the increased propensity to apoptosis in cells deficient in NOX4

**Figure 8:**
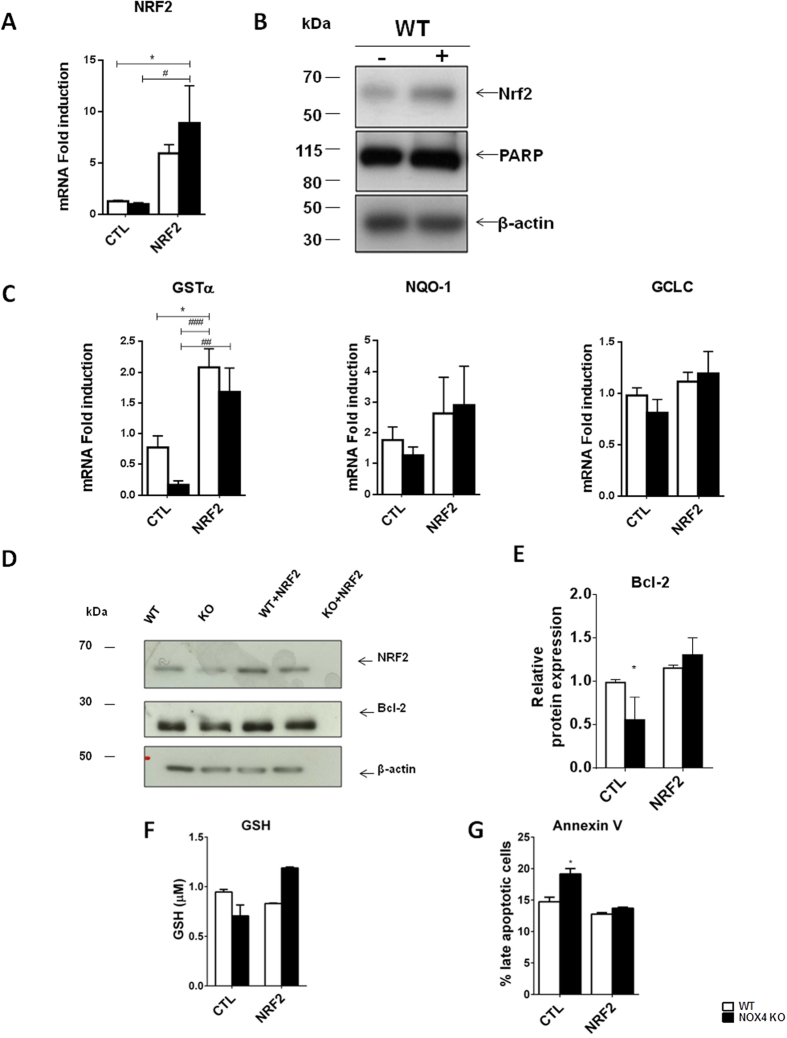
Overexpression of active NRF2 partially prevents NOX4 deletion-associated pro-apoptotic phenotype. (**A**) Real time PCR analysis of NRF2 mRNA and (**B**) Western blot analysis of NRF2 protein and β-actin performed on WT MEF cells transfected or not with active NRF2. Representative images are shown. Results are expressed as ratio of relative quantity over the mean value obtained in WT control cells ± SEM, (n = 6), ns p > 0.05, * or ^#^p < 0.05. (**C**) PCR analysis of GSTα2, NQO-1 and GCLC (**E**) mRNA expression performed on WT and NOX4 KO MEF cells transfected or not (vector) with active NRF2 (n = 6). Results are expressed as ratio of relative quantity over the mean value obtained in WT control cells ± SEM, (n = 6), ns p > 0.05, *p < 0.05, ^##^p < 0.01, ^###^p < 0.001. (**D**) Western blot analysis of NRF2, Bcl-2 and β-actin performed on WT and NOX4 KO MEF cells transfected or not with active NRF2. Representative images are shown and quantification (**E**) of Bcl-2 expression (n = 3). Results are expressed as ratio of relative quantity over the mean value obtained in WT control cells ± SEM, (n = 3), ns p > 0.05, *p < 0.05.(**F**) Measurement of total glutathione in WT and NOX4 MEF cells transfected or not with active NRF2. Results are expressed as ratio of relative quantity over the mean value obtained in WT control cells ± SEM, (n = 3), ns p > 0.05, *p < 0.05. (**G**) Annexin V/PI assay on WT and NOX4 KO MEF cells transfected or not with active NRF2. Results are expressed as percentage of late apoptotic cells in FACS analysis. (n = 3), ns p > 0.05, *p < 0.05.

We observed that NRF2 was down-regulated in the absence of NOX4. To determine whether NRF2 down-regulation plays a role in the enhanced propensity to apoptosis in NOX4 depleted cells, we transfected NOX4 KO MEF cells with an constitutively active form of the NRF2 protein^19^ (Fig. 8A,B). caNRF2 overexpression in MEF increased the expression levels of antioxidant NRF2 target gene GSTα2 whereas trends were observed for NQO1 and GCLC C (Fig. 8E). caNRF2 also rescued Bcl-2 downregulation (Fig. 8C,D). Furthermore, caNRF2 overexpression increased glutathione abundance in NOX4-deleted MEF beyond basal values of WT cells (Fig. 8F). Finally caNRF2 overexpression decreased apoptosis by Annexin V assay in NOX4 KO cells, supporting an important role for NRF2 down-regulation in the pro-apoptotic NOX4 KO phenotype (Fig. 8G).

## Discussion

In this study, we demonstrate that NOX4 deletion enhances tubular apoptosis in response to IRI in the kidney. We further show that part of this phenotype is in part related to regulation of Bcl-2 via changes in NRF2 regulation and oxidation of KEAP1. NRF2 regulation further induced alterations of glutathione cycling and mitochondrial function.

We first demonstrate that NOX4 protein increases IRI. NOX4 expression has been described to be elevated in different diseases types, but often using poorly specific antibodies^12^. Using an antibody that we characterized in NOX4 KO mice, we demonstrate that NOX4 protein expression increases 24 hours after ischemia in injured proximal tubular cells. The increased NOX4 may play a protective role in IRI. Indeed NOX4 KO mice subjected to IRI display enhanced tubular apoptosis as well as increased creatinine levels 24 hours after injury compared to controls, in conjunction with enhanced tubular lesions. This finding is consistent with our previous observation showing that NOX4 KO mice display enhanced tubular cell apoptosis in response to urinary tract obstruction^13^. In addition, a recent study shows that NOX4 promotes cell survival in stress condition via modulation of elF2α mediated signaling^40^, thereby protecting tubular cells from ER stress mediated tunicamicin apoptosis. These studies confirm that NOX4 complete deletion is detrimental *in vivo* under conditions of acute or chronic tubular injury. Since NOX4 produces low levels of ROS, mainly in the form of hydrogen peroxide, our observation is also in line with recent work demonstrating that exposure to ROS before IRI is protective^25^,^26^,^28^ and that ROS scavenging under conditions of ischemia may aggravate kidney injury^41^. Indeed, low levels of baseline ROS production may be critical for the regulation of major antioxidant pathways, such as NRF2, that will then be able to buffer acute ROS production by ischemia, likely issued in great part by mitochondria^42^.

Bcl-2 is a major anti-apoptotic protein. We observed a NOX4-dependent regulation of Bcl-2 protein in two cellular models and in mice under IRI. This regulation likely contributes to enhanced tubular apoptosis observed *in vivo* and *in vitro* and to the altered mitochondrial function^38^. Recently, activation of Bcl-2 transcription by NRF2 via ARE binding sites has been described^29^,^30^. In addition, KEAP1 may alter Bcl-2 stability^31^. Our results tend to show that NOX4 depletion regulates Bcl-2 transcription via altered NRF2 stability since transfection with an active from of NRF2 rescued this regulation *in vitro*.

As observed following unilateral urinary tract obstruction^13^, NOX4-depleted animals display decreased NRF2 protein expression as well as target gene expression. Here, we demonstrate that NOX4 modulates KEAP1 oxidation in both animals and cell lines, leading to increased NRF2 activity, and enhanced antioxidant defense. This results in decreased glutathione levels in NOX4-depleted cells *in vitro* and in animals that is rescued by NRF2 overexpression *in vitro*. Decrease in glutathione, a very potent anti-oxidant, renders kidney tubular cells vulnerable to injury under stress conditions and may disrupt several ubiquitous functions, such as mitochondrial function and cellular redox homeostasis. Previous work demonstrated that NOX4 overexpression may be deleterious for mitochondrial function and enhance apoptosis in podocytes and mesangial cells^43^. In human umbilical vein endothelial cells, NOX4 depletion was associated with stabilization of mitochondrial membrane potential and decreased H_2_O_2_ production^36^. In our experiments, embryonic fibroblasts issued from NOX4 KO mice and tubular cells depleted in NOX4 display decreased mitochondrial membrane potential, in line with decreased NRF2 expression^37^, depletion of NQO1, an important regulator of mitochondrial integrity^39^, Bcl-2 and glutathione in cells derived from NOX4 KO mice and in NOX4-depleted cells by siRNA. The enhanced propensity to tubular apoptosis of NOX4 KO mice under IRI appears related in part to regulations of major antiapoptotic and antioxidant pathways via NOX4 dependent regulation of the NRF2 pathway. Our results are consistent with published observations, including ours, that NOX4 KO mice are more susceptible to chronic tubular injury such as urinary obstruction^12^,^13^ and to tunycamycin mediated cellular injury^40^. However, they contrast with recently published observations in diabetic nephropathy where NOX4 deletion appears to be protective^14^. NOX4 expression appears to increase largely during diabetic nephropathy in podocytes and mesangial cells^44^. Therefore, it is possible that NOX4 overexpression in podocytes, where it is poorly expressed under basal conditions, is detrimental and contributes to apoptosis in conditions of hyperglycemia^45^. In mesangial cells, NOX4 accumulation may hypothetically also decrease apoptosis and lead to their accumulation, as described for lung fibroblasts^11^, although this is not demonstrated. On the other hand, NOX4 appears to be important for tubular cells, where basal expression levels are high and may participate in the global REDOX balance and pro-survival pathway regulation of these cells which are frequently subjected to stress stimuli. Since tubular cells are essential to kidney function, this pro-apoptotic effect is detrimental in conditions of acute or chronic injuries, which are relatively frequent events.

In conclusion, we demonstrate that NOX4 deletion enhances kidney tubular cell susceptibility to apoptosis by IRI, a classical model of human AKI. We further demonstrate that in the absence of NOX4, cytoprotective and antioxidant pathways are down-regulated in the kidney. NOX4 appears to be an important regulator of NRF2 in kidney tubular cells via oxidation of KEAP1 and this may explain some of the consequences of NOX4 depletion. Finally, this raises important questions regarding therapies that aim to completely abrogate ROS production in the kidney, which may alter tubular defenses in acute tubular stresses.

## Methods

### Ischemia reperfusion

WT and KO NOX4 mice on a C57BL6 background were generated as previously described^46^. Littermates were used for experiments. All animal experiments were approved by the Institutional Ethical Committee of Animal Care in Geneva and Cantonal authorities. The methods were carried out *in accordance with* the approved guidelines of the Swiss federal office for animal studies. Briefly, 22–30 gr mice mice were anesthetized by intra-peritoneal injection of ketamine and xylazine (100 mg/kg and 5 mg/kg, respectively) (GRAEUB, BayerHealthcare) and shaved. They were then laterally placed on a thermostatic working station. A 1 centimeter incision at 0.5 centimeter to the limit of the last rib and of the vertebral column was performed. The skin was separated from the muscular layer using a sterile cotton bud. A second incision of the muscular layer was performed. The kidney and renal artery were then exposed and after careful dissection, 22 minutes renal occlusion was performed using an atraumatic vascular clamp. Similar procedure was performed on the contralateral side. The animal ischemia-reperfusion and recovery was done and on a heating pad and in a thermostatic cage respectively (37 °C for both). 24 h after renal artery occlusion release, the animal was sacrificed and the two kidneys harvested for histological, biochemistry and molecular biology analysis.

### Mouse kidney histology and immunohistochemistry

Kidneys from IRI and sham operated animals were fixed in 4% paraformaldehyde (Alfa Aesar), paraffin embedded and 5 μm section cut with a microtome. Sections were stained with Haematoxylin Eosin and PAS. Immuno-histostaining were performed using citrate buffer (10 mM, pH 6) microwave based antigen target retrieval technique and the EnvisionFlex kit from Dako.

### TUNEL assay

Terminal deoxynucleotidyltransferase-mediated dUTP-fluorescein Nick End Labelling (TUNEL) was performed to detected apoptotic (necrotic) cells using DeadEnd^TM^ fluorometric TUNEL system (Promega) according to the manufacturer’s instructions. The slides were visualized and analyzed using fluorescent microscope coupled to a camera (Axiocam) and MIRAX midi slide scanning system (Zeiss) with a MIRAX viewer respectively. Quantitative analysis of TUNEL assay was carried out on slides using metamorph software. Briefly, all slides were scanned with MIRAX midi (Zeiss) coupled to Axiocam MRm (Zeiss) or photographed on a fluorescent microscope coupled to Axiocam (Zeiss). 5 random images were taken in different regions of renal cortex and Cortico medullary junction and analyzed with Metamorph image analysis software. The positive staining were matched and reported to the total tissue area of each animal under ischemia reperfusion or sham conditions. TUNEL positive cells re reported to the total tissue area respectively for apoptosis. Data were expressed as ratio of positive area by the total area examined and the mean for all animals in each group.

### Tubular injury score

Haematoxylin Eosin kidney slides were scanned and visualized with MIRAX viewer. Renal tubular injury was assessed visually by two blinded observers and average grades were computed. Tubular necrosis or tubular structural disruption was assessed and scored (0: no lesion; 1: 0–20%; 2:20–40%; 3: 40–60%; 4: 60–80%; 5: 80–100% of tubular cells showing clear morphologic alterations) at the cortico-medullary junction of each kidney.

### Serum creatinine measurement

Creatinine was measured using the Jaffe colorimetric method.

### Cell culture and transfection

mCCD_cl1_ cells were seeded and cultured as described previously^47^. WT and NOX4 KO MEF cells were seeded and cultured in complete medium (DMEM, 10%FCS and 1% penicillin streptomycin). mCCD_cl1_ and MEF Cells were seeded at low confluence (30–40%) in 6 well plates in complete medium 24 h before transfection. mCCD_cl1_ cells were transfected with 120 pmol of Stealth RNAi against NOX4 and 5 μL of lipofectamin 2000 (Invitrogen) for 72 h. The siRNA duplexes (Invitrogen) were provided as purified and annealed duplexes. The following sequences were used: siNOX4 5′-UUUAGGGACAGCCAAAUGAGCAGGC-3′ and scramble 5′-GCCACUCGUUUGUCGCCCUUGUAAA-3′. MEF cells were transfected with FuGene HD (Roche) Transfection Reagent using a 3:2 ratio (100 μL DMEM, 3 μL FuGene Transfection Reagent and 2 μg DNA) and a plasmid DNA expressing active NRF2 (a gift from Matthias Schaffer, Zurich) for 72 h. For the co-immunoiprecipitation experiments, cells overexpressing human NOX4 in an inducible fashion were used^4^. These cells express both KEAP1 and human NOX4 (data not shown).

### *In vitro* mitochondrial membrane potential assessment (Δψm) by JC-1 assay

mCCD_cl1_ and MEF cells were seeded at low confluence in 6 well-plate 24 h before JC-1 assay (Life Technologies) to assess mitochondrial membrane potential. To determine the electric potential of the inner mitochondrial membrane, cells were suspended in warm medium (DMEM phenol red free) at approximately 1 × 10^6^ cells/mL and incubated 5 minutes at 37 °C (5% CO_2_) then 30 minutes in the presence of 2 μM final of JC-1 (5′,6,6′-tetrachloro-1,1′,3,3′-tetraethylbenzimidazolylcarbocyanine iodide). 50 μM CCCP (Carbonyl cyanide -3-chlorophenylhydrazone), a mitochondrial membrane potential disrupter was used as a positive control. After the incubation, cells were pelleted, washed and suspended in 500 μL of medium before flow cytometer analysis (488 nm excitation, emission filter appropriate for Alexa Fluor 488 dye and R-phycoerythrin).

### Apoptosis assessment by Annexin V/PI assay

Cells were detached, washed with cold PBS and resuspended at 1 × 10^6^ cells/mL in 1x binding medium (10 mM Hepes [pH7.4], 140 mM NaCl and 2.5 mM CaCl_2_). Then 100 μL of the solution (10^5^ cells) were incubated with 5 μL of Annexin APC and 1 μL of PI (1 mg/mL) 15 minutes at RT (25 °C) in the dark. 400 μL of 1x binding medium were added in each tube before flow cytometer analysis.

### *In vitro* glutathione measurement by GSH-GLO assay

Cells were plated as previously described for transfection assay. Cells were then incubated 30 minutes with a GSH-GLO reagent containing a Luciferin NT-substrate and Glutathione S transferase according to the manufacturer procedure (GSH-GLO Promega). In the presence of glutathione, the GST catalyzes the reaction generating the luminogenic substrate, luciferin proportionally to the amount of glutathione. Addition of Luciferin detection reagent containing a stabilized luciferase (Ultra-Glo Luciferase) allows the luminescent reaction. The luminescent signal, proportional to the glutathione availability, is read in a 96 well plate luminometer.

### RNA extraction and Real time PCR Analysis

Total RNA from cells and kidney tissue samples was extracted using the RNA extraction kit (Machery-Nagel) according to the manufacturer’s instructions. RNA concentration and purity were measured using the NANODROP 2000 C Spectrophotometer (Thermo scientific). 0.5 to 1 μg of RNA were reverse transcripted in cDNA using the qScript cDNA SuperMix (Quanta Biosciences). 5–10ng of the cDNA were used for the real-time PCR experiment on the StepOne Plus Real-time PCR System (AB Applied Biosystems). Primers for mouse NOX4 were 5′-CCT GCT CAT TTG GCT GTC CCT A-3′ and 5′-CGG CTA CAT GCA CAC CTG AGA A-3′; primers for mouse p0 were 5′-AAT CTC CAG AGG CAC CAT TG-3′and 5′-GTT CAG CAT GTT CAG CAG TG-3′; primers for NRF2 were 5′-CTACTCCCAGGTTGCCCACA-3′ and 5′-CGACTCATGGTCATCTACAAATGG-3′; primers for GSTα2 were 5′-GCTTGATGCCAGCCTTCTG-3′ and 5′-GGCTGCTGATTCTGCTCTTGA-3′; primers for GCLC were 5′-GTTATGGCTTTGAGTGCTGCAT-3′ and 5′-ATCACTCCCCAGCGACAATC-3′; primers for NQO1 were 5′-GAGCTTTAGGGTCGTCTTGG-3′ and 5′-AGGATCGTAATACCGAACGC-3′; primers for TRX were 5′-GATGCACCAGGCAGCTTTG-3′ and 5′-TCTTCGACTTTCCAGCCATAGT-3′; primers for Calreticulin were 5′-TCCGGTGTAAGGATGATGAA-3′ and 5′-AGTCCCAATCATCCTCCAAG-3′; primers for BIP were 5′-AGGAGACTGCTGAGGCGTAT-3′ and 5′-CAGCATCTTTGGTTGCTTGT-3′; primers for GRP94 were 5′-TTGAACCTCTGCTCAACTGG-3′ and 5′-ATCCATACTGACTGGCCACA-3′; primers for CHOP were 5′-GGAAACGGAAACAGAGTGGT-3′ and 5′-TCCTGCTCCTTCTCCTTCAT-3′.

### Western blot analysis

Cells or kidney tissue samples were homogenized in 100 μl or 1 ml respectively in cold lysis buffer (20 mmol/L Tris-HCl; 2 mmol/L EDTA; 30 mmol/L NaF; 30 mmol/L NaPPi; 0.5 mol/L Na_3_VO_4_; 20% SDS; 10% Triton-X-100 and Roche-Complete mini protease inhibitor mixture) on ice. Protein concentration assay was performed using both BCA protein Assay (Thermo Scientific, PIERCE) and Coomassie Blue gel staining (Thermo Scientific GelCode blue stain Reagent). For protein oxidation analysis in non-reduced condition, lysis buffer is completed with 40 mM of NEM (N-ethylmaleinide) in absence of β-mercaptoethanol. Equal amount of protein were separated by 4–12% Bis-Tris pre Cast protein Gel (Invitogen) or 10% SDS-PAGE gel and transferred to polyvinylidene difluoride membranes (Immobilon-P, Milipore). Rabbit polyclonal anti-PARP (Cell Signaling), Rabbit anti-NRF2 (Santa Cruz), Rabbit anti-Keap1 (Proteintech), Rabbit anti Bcl-2 (Cell signaling and Santa Cruz), Mouse anti-glutathionylated proteins (Virogen) and mouse anti-β-actin (Sigma) antibodies were used. HRP labeled polyclonal anti-mouse or rabbit Ig (BD Biosciences Pharmingen) were used and the antigen-antibody complexes were detected by the chemiluminescent HRP substrate method (Immobilon, Milipore). Bands were quantified using a Java-based image processing software (ImageJ). A rabbit monoclonal antibody against NOX4 was used as described^48^

### Freshly Isolated Renal Cortical Tubule Suspensions

Renal cortex was isolated using a modification of the method described by Guder^49^ previously described by Fenton^50^). Cortex from both kidneys was dissected, sliced into ∼1-mm pieces, and then placed in an enzyme solution containing 0.5 mg collagenase type IIand 0.5 mg/mL protease inhibitor pronase (Roche Diagnostics) at 37 °C in buffer B (125 mM NaCl, 0.4 mM KH2PO4 1.6 mM K2HPO4, 1 mM MgSO4, 10 mM Na-acetate, 1 mM α-ketogluterate, 1.3 mM Ca-gluconate, glucose 30 mM, 5 mM glycine, 48 μg/mL trypsin inhibitor, and 25 μg/mL DNase, pH 7.4). Two milliliters of enzyme solution was used for each kidney cortex. Samples were mixed continuously at 850 × g at 37 °C. After 10 min, half of the enzyme solution was removed and replaced with buffer B, and samples were incubated for a further 10 min. This procedure was repeated for another 10 min. Samples were then spun at 200 × g for 2 min, buffer B was removed and samples were resuspended in HamF12/DMEM cell medium. The cortical tubular suspensions were kept at 37 °C under 5% CO_2_ and 19% O2 during 4 hours.

### Co-Immunoprecipitation

Co-Immunoprecipitation was performed as previously described^51^. In brief, cells were harvested under non denaturing conditions with 500 uL of RIPA buffer and then centrifuge for 15 minutes at 13,000 RPM, 4 °C. A pre-clearing step was conducted on the resulting supernatant with 100 μl of GammaBind Plus Sepharose beads for 60 minutes, 4 °C. The primary antibody was incubated on a tube roller for 3 hours at 4 °C. GammaBind Plus Sepharose beads were added for another 30 minutes at 4 °C with gentle rocking. After a 2 min centrifugation at 5000 RPM, 4 °C the protein pellet was re-suspended with 50 μl of SDS-PAGE sample buffer for later use.

### Statistics

Groups statistics were analyzed by t test and two way ANOVA/Bonferroni’s Multiple Comparison Test respectively for two or multiple groups.

## Additional Information

**How to cite this article**: Nlandu-Khodo, S. *et al*. NADPH oxidase 4 deficiency increases tubular cell death during acute ischemic reperfusion injury. *Sci. Rep.* **6**, 38598; doi: 10.1038/srep38598 (2016).

**Publisher's note:** Springer Nature remains neutral with regard to jurisdictional claims in published maps and institutional affiliations.

## Supplementary Material

Supplementary Figures and Legends
