# Supplementary material for: NADPH oxidase 4 deficiency increases tubular cell death during acute ischemic reperfusion injury
Source: Sci Rep. 2016 Dec 7;6:38598. doi: 10.1038/srep38598 (PMC5141508; doi:10.1038/srep38598)
Supplement: Supplementary Figures and Legends [file srep38598-s1.pdf]

NADPH oxidase 4 deficiency increases tubular cell death during acute ischemic reperfusion injury

Stellor Nlandu-Khodo*[1], Romain Dissard*[1], Udo Hasler[1], Matthias Schäfer[2], Haymo Pircher[3], Pidder Jansen-Durr[3], Karl Heinz Krause[4] ,Pierre-Yves Martin[1] and Sophie de Seigneux[1]

Supp. Figure 1

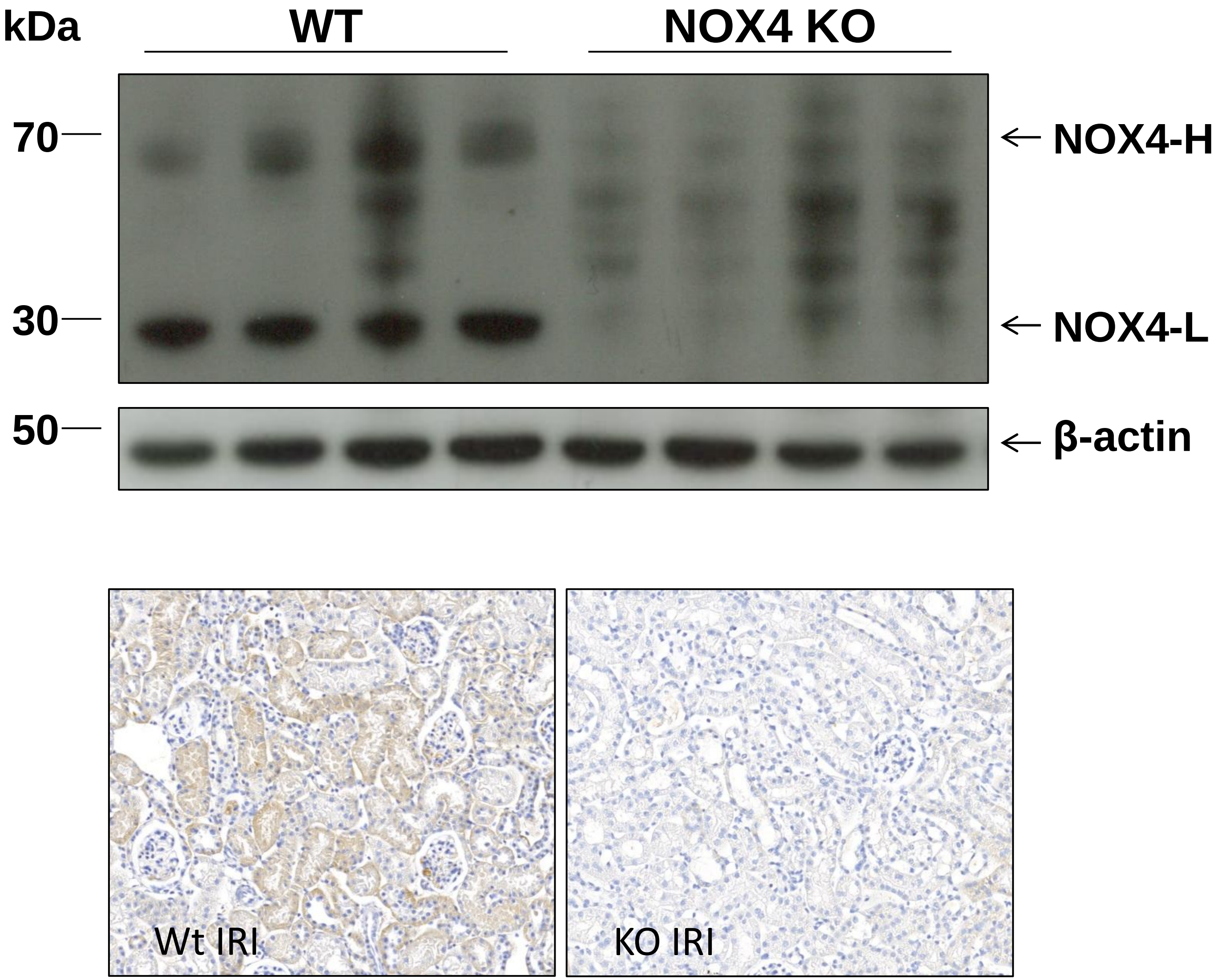

Supp. Figure 2

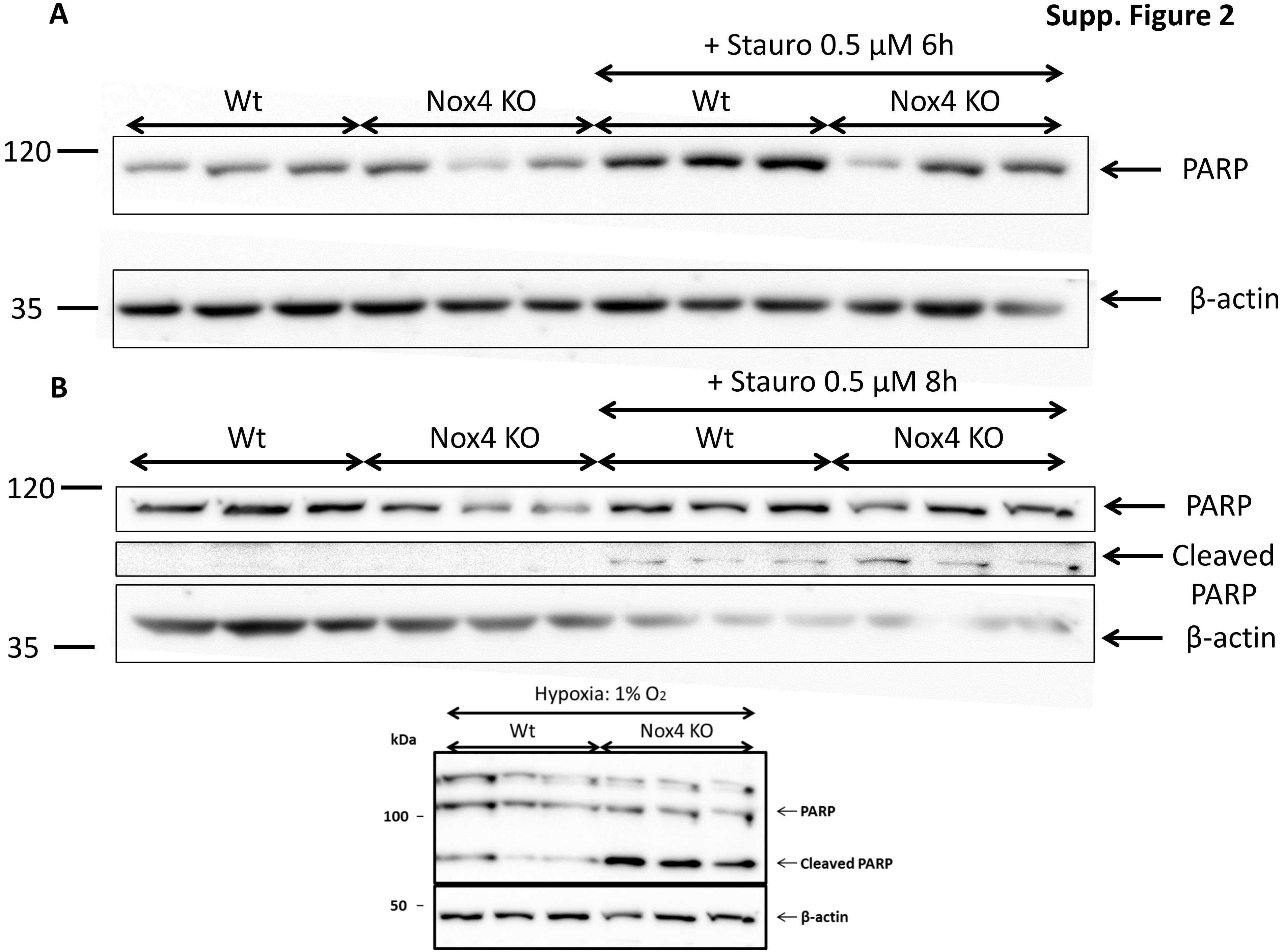

Supp. Figure 3

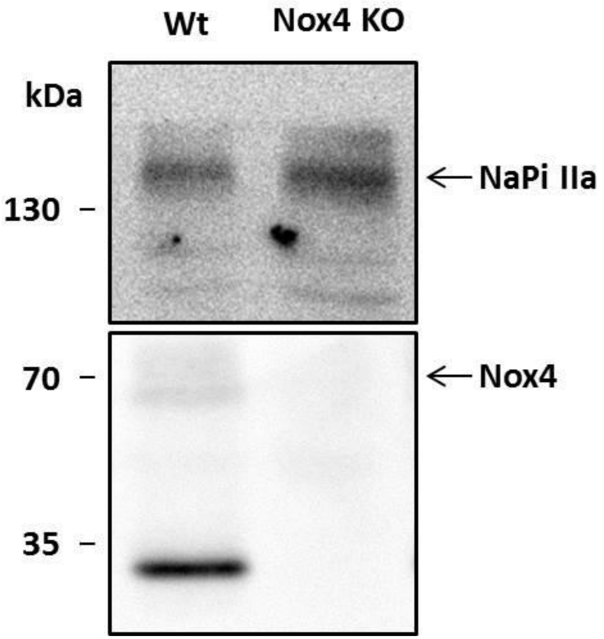

Supp. Figure 4

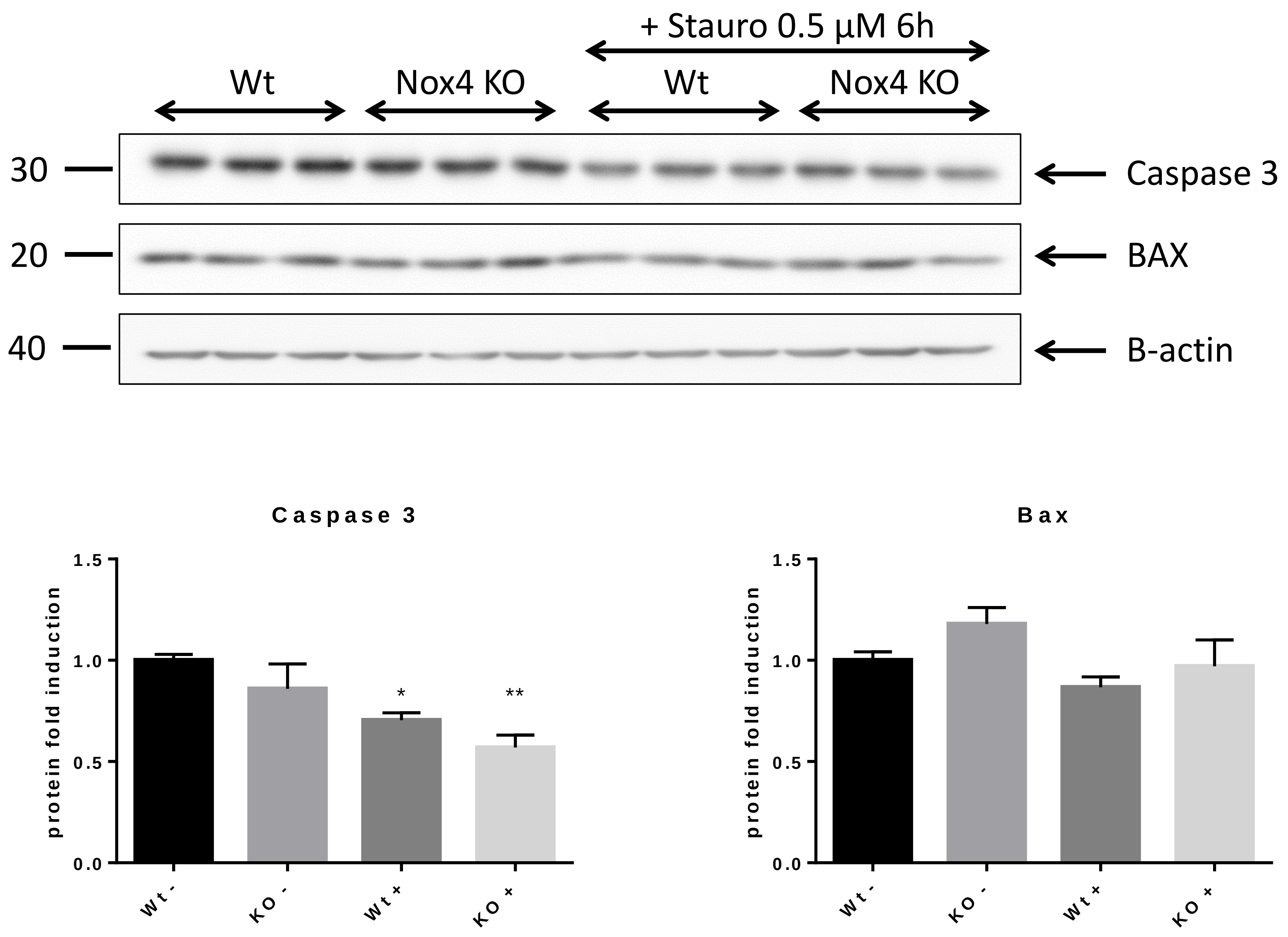

Supp. Figure 5

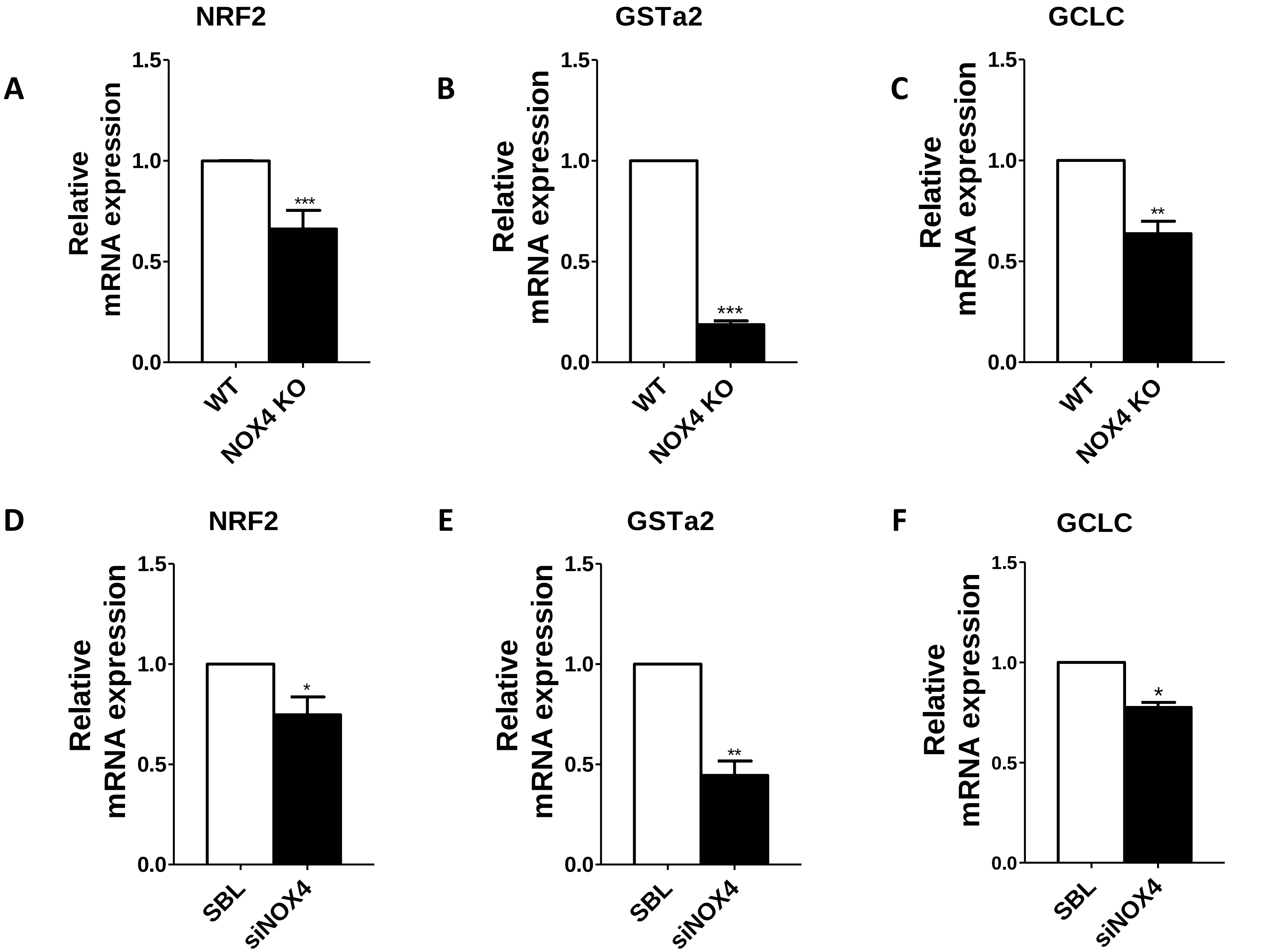

Supp. Figure 6

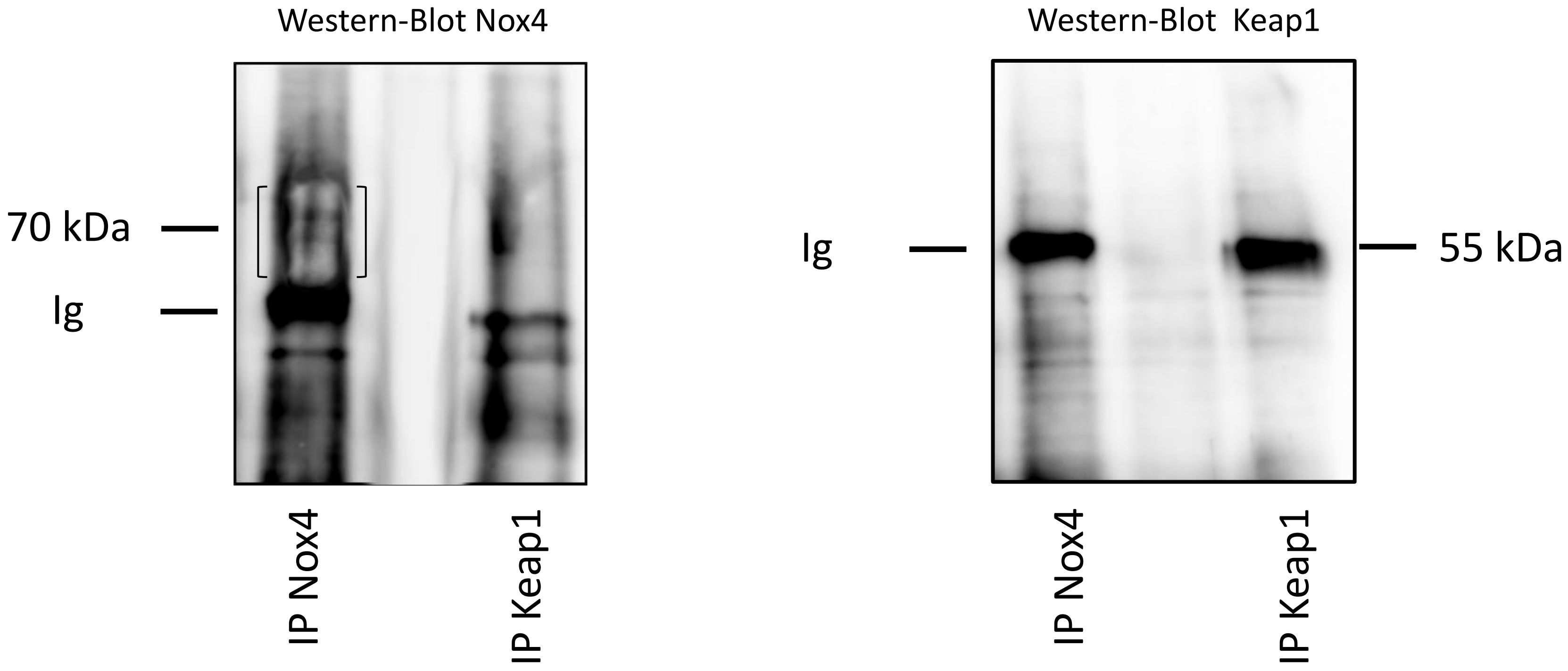

Supp. Figure 7

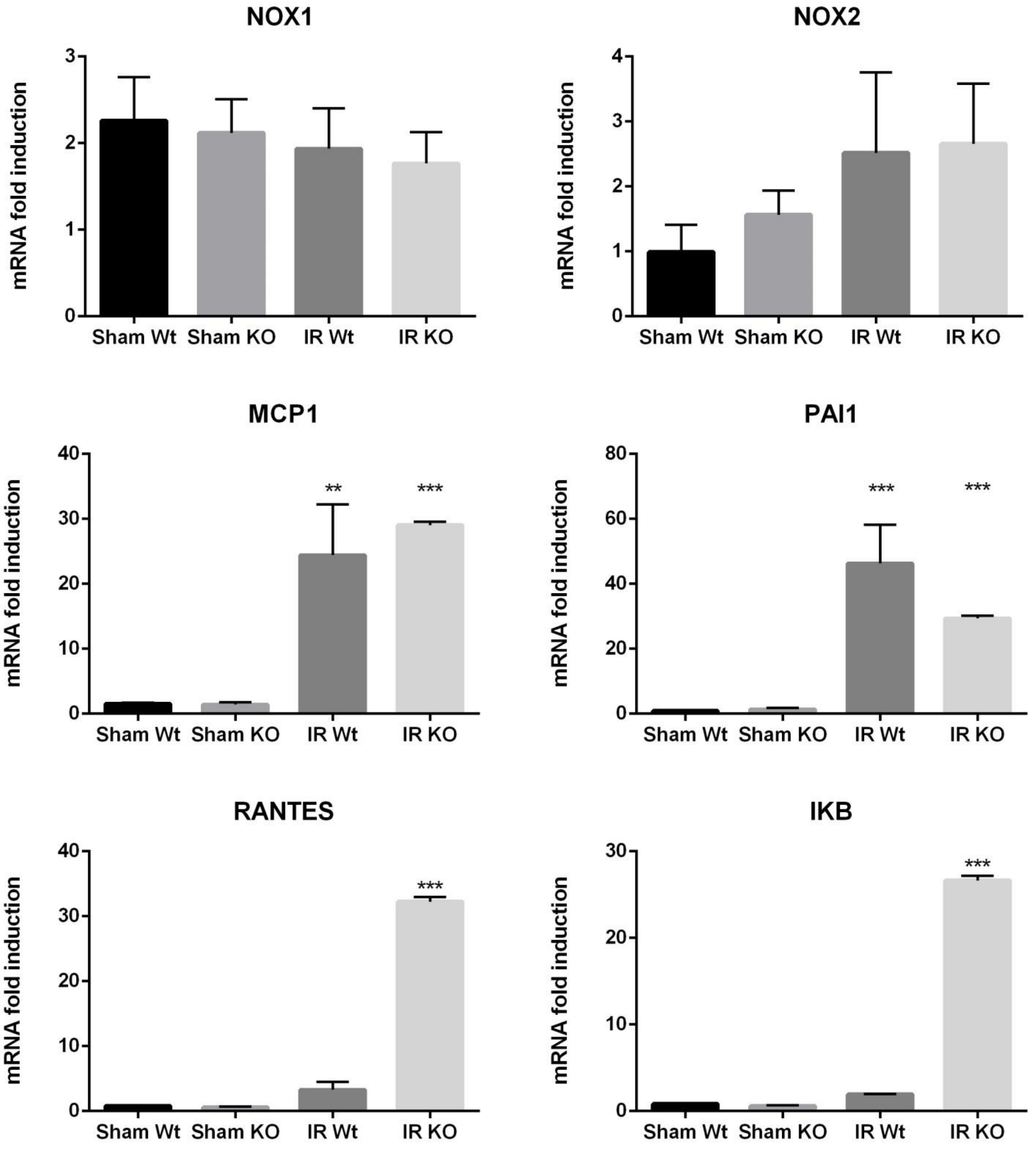

Supp. Figure 8

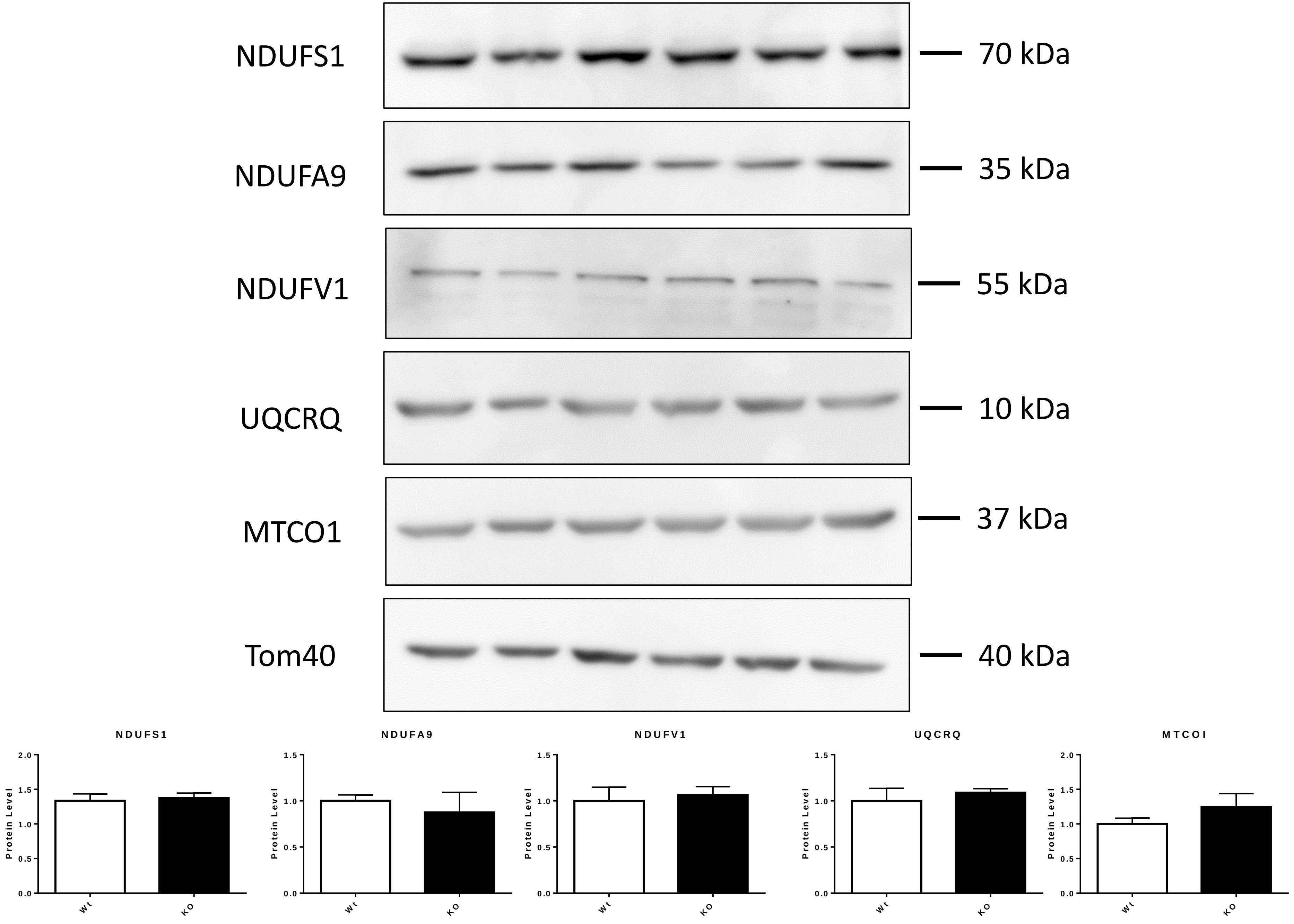

Supplementary Figure 1: Characterization of NOX4 antibody

NOX4 Western blot analysis performed on WT and NOX4 KO animals. Both canonical NOX4 (NOX4-H, 65 kDa) and the lower molecular weight isoform (NOX4-L, 28 kDa) were absent in NOX4 KO animal kidneys. Representative images of NOX4 immunostaining performed in the cortex of IRI kidneys from WT (left) and NOX4 KO (right) mice.

Supplementary Figure 2: PARP Western blot analysis performed on WT and KO NOX4 MEF cells treated or not with staurosporine. Full length PARP was downregulated at 6 (A) and 8 (B) hours in KO NOX4 MEF cells. Cleaved PARP isoform increased in cells treated by Staurosporine with a larger expression in KO NOX4 MEF cells compared to WT. Full length PARP was also downregulated after 4 hours of 1% oxygen hypoxia in KO NOX4 MEF cells. Cleaved PARP isoform increased in KO NOX4 MEF submitted to hypoxia (C).

Supplementary Figure 3: NaPi IIa is detected in both the freshly isolated renal cortical tubule suspensions from Wt and KO NOX4 mice. The sodium/phosphate cotransporter or NaPi IIa is a protein found in the proximal tubule of the nephron. Its detection is the proof of concept that the suspension contains tubules. Both canonical NOX4 (NOX4-H, 65 kDa) and the lower molecular weight isoform (NOX4-L, 28 kDa) were absent in freshly isolated renal cortical tubule suspensions from NOX4 KO animal.

Supplementary Figure 4: Caspase 3 and Bax Western blot analysis performed on WT and KO NOX4 MEF cells treated or not with staurosporine, ns $p>0.05$, * $p<0.05$, **$p<0.01$

.**Supplementary Figure 5**: NRF2 and NRF2 target gens are down-regulated in MEF cells derived from NOX4 KO mice (A-C) and $mCCD_{cl1}$ cells with silencing of Nox4 (D-F) Real time PCR analysis of NRF2 (A and D), GSTα2 (B and E), GCLC (C and F) performed on MEF cells derived from WT and NOX4 KO animals showing a defective antioxidant system

in line with previous data observed in $mCCD_{cl1}$ renal cells. (n=3), ns p>0.05, * p<0.05, **p<0.05

Supplementary Figure 6: Western-Blot analysis Of NOX4 and Keap1 immunoprecipitation in Cells overexpressing human Nox4. Keap1 size precluded its visualization in a co-IP.

Supplementary Figure 7: Real time PCR analysis of NOX1, NOX2, MCP1, PAI1, RANTES and IkB performed on the kidney tissue of Wt and KO NOX4 mice under ischemic condition (Sham operated as inner control). There is no significant variation of the expression of NOX1 but there is nevertheless an increased trend for NOX2 under ischemic conditions. We observed statistical difference in MCP1, PAI1, RANTES and IkB that are key actors of the inflammatory response. (n=5), ns p>0.05, * p<0.05, ** p<0.01, *** p<0.001.

Supplementary Figure 8: Western blotting and quantification of Complex I (NDUFS1, NDUFA9, NNDUFV1), III (MTO1), IV (UQCRQ) mitochondrial subunits., in WT and NOX4 KO MEF cells .
